# R-Spondin 1 (RSPO1) Increases Mouse Intestinal Organoid Unit Size and Survival *in vitro* and Improves Tissue-Engineered Small Intestine Formation *in vivo*

**DOI:** 10.3389/fbioe.2020.00476

**Published:** 2020-06-05

**Authors:** Gabriel Levin, Samuel M. Zuber, Anthony I. Squillaro, Mari Cleide Sogayar, Tracy C. Grikscheit, Ana Claudia O. Carreira

**Affiliations:** ^1^Cell and Molecular Therapy Center (NUCEL), School of Medicine, University of São Paulo, São Paulo, Brazil; ^2^Interunits Graduate Program in Biotechnology, Institute of Biomedical Sciences, University of São Paulo, São Paulo, Brazil; ^3^Developmental Biology and Regenerative Medicine Program, Saban Research Institute, Children's Hospital Los Angeles, Los Angeles, CA, United States; ^4^Biochemistry Department, Chemistry Institute, University of São Paulo, São Paulo, Brazil; ^5^Department of Surgery, School of Veterinary Medicine and Animal Science, University of São Paulo, São Paulo, Brazil

**Keywords:** recombinant human R-Spondin 1 (rhRSPO1), tissue-engineered small intestine (TESI), stem cells, organoid unit (OU), 3D culture, scaffold, tissue engineering

## Abstract

**Introduction:** Cell therapy and tissue engineering has recently emerged as a new option for short bowel syndrome (SBS) treatment, generating tissue engineered small intestine (TESI) from organoid units (OU) and biodegradable scaffolds. The recombinant human R-Spondin 1 (rhRSPO1) protein may be a key player in this process due to its mitogenic activity in intestinal stem cells.

**Objective:** Aiming at optimizing the TESI formation process and advancing this technology closer to the clinic, we evaluated the effects of rhRSPO1 protein on OU culture and TESI formation.

**Methods:** Intestinal OU were isolated from C57BL/6 mice and cultured in Matrigel in the presence or absence of recombinant human rhRSPO1. Throughout the culture, OU growth and survival rates were evaluated, and cells were harvested on day 3. OU were seeded onto biodegradable scaffolds, in the presence or absence of 5 μg of rhRSPO1 and implanted into the omentum of NOD/SCID mice in order to generate TESI. The explants were harvested after 30 days, weighed, fixed in formalin and embedded in paraffin for histological analysis and immunofluorescence for different cell markers.

**Results:** After 3 days, rhRSPO1-treated OU attained a larger size, when compared to the control group, becoming 5.7 times larger on day 6. Increased survival was observed from the second day in culture, with a 2-fold increase in OU survival between days 3 and 6. A 4.8-fold increase of non-phosphorylated β-catenin and increased relative expression of *Lgr5* mRNA in the rhRSPO1-treated group confirms activation of the canonical Wnt pathway and suggests maintenance of the OU stem cell niche and associated stemness. After 30 days of *in vivo* maturation, rhRSPO1-treated TESI presented a larger mass than constructs treated with saline, developing a more mature intestinal epithelium with well-formed villi and crypts. In addition, the efficiency of OU-loaded rhRSPO1-treated scaffolds significantly increased, forming TESI in 100% of the samples (*N* = 8), of which 40% presented maximum degree of development, as compared to 66.6% in the control group (*N* = 9).

**Conclusion:** rhRSPO1 treatment improves the culture of mouse intestinal OU, increasing its size and survival *in vitro*, and TESI formation *in vivo*, increasing its mass, degree of development and engraftment.

## Introduction

Short bowel syndrome is the most common type of intestinal failure, affecting both adults and children, manifesting as a result of 50–75% loss of the intestinal surface, either by surgical resection or congenital malformation (Thompson, 1994; Stollman et al., 1996; Spencer et al., 2005; O'Keefe et al., 2006; Beattie et al., 2010; Thompson et al., 2012). This intestinal loss leads to poor nutrition and fluid absorption, resulting in weight loss, electrolyte imbalance and other symptoms (O'Keefe et al., 2006). Accurate data regarding the incidence and prevalence of SBS are elusive because of the lack of consensus and precision in definition of this syndrome (O'Keefe et al., 2006). Although SBS has a significant prevalence in adults, among children, the situation is even more serious, with a 3-year median survival rate of only 60% after transplantation (Kato et al., 2006). A group of researchers suggest that the incidence of SBS is ~24.5 cases per 100,000 newborns (Wales et al., 2004), while another study points to an incidence close to 1% (Cole et al., 2008).

Despite the existence of therapies to improve SBS symptoms, no curative treatment is available and, in many cases, surgical treatment is unavoidable, often resulting in lifelong dependence on parenteral nutrition. These surgeries involve complex procedures associated with high morbidity and mortality rates in children and adults and high costs, depending, in addition, on the availability of organs for donation, organ compatibility and immunosuppressive drugs administration (Reyes et al., 1998; Messing et al., 1999; Grant et al., 2005; Kato et al., 2006; Bharadwaj et al., 2017). In this context, cell therapy with stem cells and tissue engineering emerge as a new therapeutic option for several intestinal disorders, generating tissue engineered small intestine (TESI) for autologous transplantation. Several approaches have been developed to generate TESI, the main one being based on a biodegradable polymeric scaffold seeded with intestinal organoid units (OU), implanted in a vascularized region for *in vivo* maturation (Grikscheit et al., 2004; Sala et al., 2009; Barthel et al., 2012; Spurrier and Grikscheit, 2013; Finkbeiner et al., 2015; Grant et al., 2015; Hou et al., 2018).

Organoid units (OU) are multicellular clusters containing a lumen, composed of an epithelium and mesenchyme, and capable of secreting important signaling factors for the maintenance of these structures (Hou et al., 2018). Among other things, the success of this technique is based on the OU ability to populate the scaffold with proliferative cells that will, in the resulting TESI, contain different intestinal cell types, recapitulating the native organ architecture, organization, and regenerative dynamics. Unlike cultures of Lgr5^+^ cells or crypts, the mesenchyme-containing OU survive *in vitro* without the addition of signaling factors, such as EGF, R-Spondin1, Dll4, Noggin, or Wnt3a (Watson et al., 2014; Hou et al., 2018). However, the supplementation with peptide factors may improve OU survival and TESI formation, as observed for enteroids (Sato et al., 2009, 2011; Matthews et al., 2011; Abo and Clevers, 2012; Schuijers and Clevers, 2012; Mahe et al., 2013; Belchior et al., 2014; Watson et al., 2014), improving its functionality.

The intestinal mucosa epithelium is composed mainly of four different specialized cell types, namely: enterocytes, Paneth cells, goblet cells, and enteroendocrine cells (van der Flier and Clevers, 2009; Schuijers and Clevers, 2012). These cell types differentiate from the Lgr5+ intestinal stem cells as the daughter cells distance themselves from the bottom of the crypt in a very dynamic process along the crypt-villus axis, undergoing apoptosis as they reach the apex of the villus. Intestinal villi protrude into the mucosa toward the lumen and are composed predominantly of enterocytes (absorptive cells), goblet cells (mucus-secreting cells), and enteroendocrine cells (hormone secreting cells). On the other hand, the crypts are invaginations of the epithelium, which depart from the base of the villi and mature by traveling in the opposite direction. The base of the crypt is inhabited by intestinal stem cells (ISCs) and Paneth cells, which, respectively, ensure regeneration of the epithelium by producing new cells and maintain the stem cell niche (Day, 2006; Barker et al., 2007; van der Flier and Clevers, 2009; Sato et al., 2011; Schuijers and Clevers, 2012).

These techniques for generating TESI might augment treatment of SBS. To ensure the functionality of TESI, it is necessary to form a complete intestinal epithelium, as well as an associated innervated muscular layer, promoting coordinated peristalsis and adequate absorption of nutrients and fluids. Although OU can be cultured and generate TESI containing all of these elements without the addition of exogenous growth and differentiation factors (Sala et al., 2011; Hou et al., 2018), it is desirable to optimize this process to form larger and more efficient functional tissues for future human therapies. In this context, the recombinant human R-Spondin 1 (rhRSPO1) protein, given its mitogenic activity in intestinal stem cells, along with other peptide factors, may be key players to improve the TESI formation, development and functionality.

RSPO1 (roof plate-specific Spondin1) is a secreted protein known to induce proliferation of ISCs through activation of the Wnt pathway, with insignificant effects on differentiated cell migration and differentiation along the crypt-villus axis (Kim et al., 2005). The therapeutic application of RSPO1 has already been explored in animal models for treatment of intestinal inflammatory diseases (Kim et al., 2005; Zhao et al., 2007) and mucositis, induced by chemotherapy (van Vliet et al., 2010) or radiotherapy (Ley et al., 2017), indicating beneficial effects. *In vitro*, RSPO1 allows continuous propagation of intestinal organoids that lack mesenchyme (Ootani et al., 2009; Sato et al., 2009; Barker et al., 2010), allowing *ex vivo* applications. However, for construction of an adequate TESI for SBS curative therapy in humans, it is essential to increase the engineered intestinal surface area and to include cells such as the muscle and enteric nervous system that a functional intestinal replacement would require. Therefore, rhRSPO1 is expected to improve the process of TESI formation, given its capacity to induce intestinal stem cells proliferation, respecting the natural order of tissue self-renewal events (Abo and Clevers, 2012). In the present work, the biological activity of recombinant human RSPO1 protein, produced with a heterologous human cell line system (Levin et al., 2020), was assessed in cultures of murine organoid units and TESI formation to optimize the development of this process.

## Materials and Methods

### Animals

All experiments involving mice were carried out according to the protocols approved by the IACUC of Children's Hospital Los Angeles, which adheres to the US National Institute of Health's Office of Laboratory Animal Welfare Guidelines (Approval #215). C57BL/6 wild type mice (000664—The Jackson Laboratory), Actin^egfp^ (Sala et al., 2011), Lgr5^egfp^ (008875—The Jackson Laboratory), and non-obese diabetic/severe combined immunodeficient mice (NOD/SCID, 005557—The Jackson Laboratory) were maintained on C54BL/6 background.

### Intestinal Organoid Unit Isolation

The OU isolation process was adapted from our previously published protocol (Sala et al., 2011; Grant et al., 2015; Hou et al., 2018). Two-week-old C57BL/6 mice were euthanized in a CO_2_ chamber and the small intestine was collected and opened longitudinally. The resected tissue was vigorously washed five times with cold Hank's Balanced Salt Solution (HBSS, 14170-112, Life Technology) containing Antibiotic/Antimycotic (Anti/Anti, 15240062, Life Technology) to remove the intestinal content. The intestines were minced into 1 × 1 mm^2^ pieces, washed twice in cold HBSS/Anti/Anti, sedimented at 164 g (Eppendorf 5810R−1,000 rpm) between washes, and digested with type IV collagenase (800 units/ml, CLS-4, Worthington) and dispase (0.12 mg/ml, 17105-041, Gibco) in HBSS for 20 min at 37°C. The digestion was stopped with 10% fetal bovine serum (FBS, Invitrogen) in high-glucose Dulbecco's modified Eagle's medium (DMEM, 10566-016, Invitrogen), the sediment (pellet) was minced with a pipette and centrifuged at 41 g (Eppendorf 5810R−500 rpm) for 5 min to remove single cells. The resulting pellet containing the OU was resuspended in DMEM supplemented with 10% FBS, non-essential amino acids (NEAA, 11140-076, Life Technology) and Anti/Anti, centrifuged one last time at 105 g (Eppendorf 5810R−800 rpm) and the supernatant was removed.

### OU Culture and Quantification of Size and Survival Rate

The OU pellet was resuspended in four times the volume of DMEM/10% FBS/Anti/Anti/NEAA, mixed in equal parts with reduced growth factor Matrigel matrix (354230, Corning) and seeded onto adherent cell culture plates, forming a three-dimensional environment (3D) for OU growth. Two different experiments were carried out to assess the survival rate and growth of the OU. Forty microliters of the OU suspension in Matrigel matrix were plated onto 24-well plates for the survival rate experiment and 60 μL were plated onto 12-well plates for the growth experiments. The plates were incubated at 37°C for 20 min for Matrigel polymerization, and culture medium (DMEM/10% FBS/Anti/Anti/NEAA), containing rhRSPO1 (500 ng/mL) (Levin et al., 2020), or phosphate buffered saline (PBS) (control group), was added to each well. OU cultures were maintained in an incubator at 37°C and 5% CO_2_ with medium change every 2 days. Preliminary dose standardization of rhRSPO1 was performed by comparing two concentrations: 500 and 200 ng/mL (Supplementary Figure 1).

To assess the survival rate of OU in culture, the number of OU found in four random fields per well, identified at the bottom of the well, was counted along the z axis every day over 6 days, under a light microscope. For each condition, the survival rate was indirectly calculated from the ratio between the mean number of OU on each day by the number of OU on the first day (OU number each day/OU number on Day 1). To assess OU growth in culture, eight random fields per well were imaged along the z axis under a Leica microscope (DMI6000B) on days 1, 3, and 6, and the cross-sectional area of each OU was determined with ImageJ software. The average area of all identified organoid units in the eight random fields selected in each well was considered a technical replicate. Three biological replicates, with three technical replicates per condition, were adopted for each experiment.

### Harvest and Storage of *in vitro* Grown OU for Analysis

Organoid units grown *in vitro* were collected on days 3 and 6 of culturing for qRT-PCR, Western blot and Histological analysis, respecting the appropriate form of harvesting and storage for each material. It is worth mentioning that only the OU from plates where there was no unnecessary stress to the cells were collected, preserving their integrity for WB and qRT-PCR analyses. To this end, Matrigel containing the OU was collected from wells in 15 ml conical tubes and centrifuged at 193 g (Rcf) (Eppendorf 5810R) for 5 min for sedimentation. For sample preparation for histological analysis (Hematoxylin & Eosin (H&E) and Immunofluorescence), the OU-containing pellet was soaked in 3% low melting agarose in PBS, then fixed overnight in 10% buffered formalin (23-305-510, Fisher HealthCare) for paraffin embedding, as described in the literature (Hou et al., 2018). For total RNA extraction and qRT-PCR analysis experiments, the OU-containing pellet was placed in RNA Later (AM7021, Lifetech) and stored at −80°C. For total protein quantification and Western blot experiments, cell extracts were prepared from the OU pellet in RIPA^+^ buffer [150 mM NaCl, 50 mM Tris, 1 mM EDTA, 1 mM EGTA, 1% Triton X-100, 0.1% SDS, 0.5% sodium deoxycholate, pH 7.4 + Halt cocktail protease inhibitor (1:100; P87786, Thermo-Fischer) + cocktail phosphatase inhibitor 2 (1:100; P5726, Sigma-Aldrich) + cocktail phosphatase inhibitor 3 (1:100; P0044, Sigma-Aldrich)]. Prior to storage at −80°C, protein extract aliquots intended for Western blot experiments were previously denatured by mixing in denaturing loading buffer containing β-mercaptoethanol and incubating samples at 98°C for 5 min.

### qRT-PCR Analysis

Total RNA from OU samples treated with 500 ng/mL of rhRSPO1 or PBS at day 3 of culture was extracted with the RNeasy Mini Kit (74134, Qiagen). Five hundred nanograms total RNA were processed for cDNA synthesis with the iScript™ Reverse Transcription Supermix for RT-qPCR (1708841, Bio-Rad). The relative mRNA expression levels of *cyclin D1, Lgr5*, and β*-Catenin* were assessed with the LightCycler® 480 SYBR Green I Master in a LightCycler® 480 System (04707516001, Roche). All steps followed the manufacturer's instruction. A dissociation cycle was run after each cycle to check for non-specific amplification. Relative mRNA expression levels were estimated based on the delta delta Ct (ΔΔCt) method (Livak and Schmittgen, 2001), with *Gapdh* as the reference housekeeping gene for normalization. All primers (Eurofins Genomics) are listed in Supplementary Table 1.

### Western Blot Analysis

The amount of total protein in each RIPA^+^ buffer cell extract from the third day of OU culture (Day 3), treated with 500 ng/mL rhRSPO1 or PBS (control), was quantified by the Bio-Rad DC Protein assay (Bio-Rad). The Western blot analysis was carried out with Thermo Fischer Scientific Western Blot System. To this end, 20 μg of total protein, previously mixed with loading buffer (containing β-mercaptoethanol), were applied to the polyacrylamide gel (NW00080BOX, Invitrogen Bolt 8% Bis-Tris Plus). After electrophoresis, the proteins were transferred to the nitrocellulose membrane with the iBlot™ Transfer Stack system (IB301002, Thermo Fischer Scientific) and non-specific sites were blocked with non-fat milk (5%). Specific stainings were carried out with the following antibodies: anti-non-phospho (active) β-catenin (1:1,000; #8814, Cell Signaling) and anti-β-actin (1:1,000; SC-69879, Santa Cruz Biotechnology), anti-rabbit IgG HRP linked (1:1,000; #70745, Cell Signaling), and anti-mouse IgG HRP linked (1:1,000; #70746, Cell Signaling). All antibodies were diluted in blocking solution (non-fat milk 5%) and are listed in Supplementary Table 2. Immobilon Western Chemiluminescent HRP Substrate (WBKLS0500, Millipore) was added to the membrane at the time of exposure in the C-DiGit Chemiluminescent Western Blot Scanner (LI-COR). Quantitative densitometric analysis of the Western blot membrane was carried out with the ImageJ software. The final result of β-catenin expression was generated after normalization relative to the β-actin reference protein.

### OU Implantation and TESI Formation

OU were seeded onto biodegradable scaffolds and implanted into the omentum of adult NOD/SCID recipient mice to generate TESI, as previously described (Sala et al., 2011). Cylindrical scaffolds of PGA/PLLA (poly-glycolic acid/poly L-lactic acid) of 5 mm length, prepared as described in a previous work by our group (Sala et al., 2011), were loaded with 60 μl of OU pellet and maintained on ice until the time of implantation. To evaluate the influence of rhRSPO1 on TESI formation, each scaffold + OU complex was treated with either: a total of 5 μg of rhRSPO1 or the same volume of PBS (control). Half of the protein was seeded onto the scaffold, while the remaining half was directly mixed into the OU aliquot. The OU-loaded scaffolds were implanted into the omentum of NOD/SCID recipient mice and allowed to mature *in vivo* for 1 month (30 days). The *in vivo* matured OU-loaded scaffolds were then explanted, carefully dissected to remove adjacent tissue, weighed on an analytical scale (AB204-S, Mettler Toledo) and fixed in formalin for paraffin embedding. During animal necropsy, adjacent tissues were searched for the presence of tumors.

### TESI Complexity Evaluation

Histological sections from the TESI samples, cross-sectioned following the same orientation, were stained with H&E and analyzed under a phase contrast microscope to assess the level of organization of the engineered intestinal epithelium. For comparison purposes, the tissues were independently evaluated with respect to their degree of maturation and classified by three different scientists with previous experience in observing engineered intestinal epithelium. The samples were classified based on the level of complexity of the TESI epithelium (Supplementary Figure 2), being divided into: Low complexity—poorly developed epithelium in the form of rosettes (A); Medium-low complexity—continuous linear epithelium (B); Moderate complexity—continuous epithelium with villus-like and rudimentary crypt structures (C); High complexity—continuous epithelium with well-defined crypts, villi and clear presence of secretory cells (D). The final results of the levels of development of the engineered intestinal epithelium of the samples from each one of the groups, were generated from the consensual classification obtained by all three independent histological evaluations.

### Immunostaining

Five micrometers-thick sections of paraffin-embedded tissues, cross-sectioned following the same orientation, were placed onto positively charged microscope slides. The sections were deparaffinized in HistoChoice Clearing Agent (H2779, Sigma-Aldrich) solution and incubated with decreasing concentrations of ethanol until fully rehydrated. The antigen retrieval step was carried out with citrate-based antigen unmasking solution (H3300, Vector Labs) or Tris-based unmasking solution (H3301, Vector Labs), while heating in the microwave before cooling to room temperature and washing in PBS-T (PBS with 0.1% Tween 20). Non-specific sites were blocked with Universal Blocking Solution (UBS, 1% BSA, 0.1% cold fish skin gelatin, 0.5% Triton-X 100 and 1x PBS) supplemented with 2% donkey serum (D9663, Sigma-Aldrich). The tissues were incubated overnight at 4°C with different primary antibodies, diluted in UBS + 2% donkey serum.

Immunostaining was carried out with the following primary antibodies: Chromogranin A (ChGA, 1:400; ab15160, Abcam), E-Cadherin (1:100; 610181, BD), GFP (1:200; ab290, Abcam), Lysozyme (1:100; A0099, DAKO), Mucin2 (1:100; sc15334, Santa Cruz), PCNA (1:400; ab29, Abcam), Smooth Muscle D-Actin (SMA, 1:100; ab5694, Abcam), Tuj1 (1:1,000; 801202, BioLegend), and Villin (1:100; sc-66022, Santa Cruz). For detection, the tissues were incubated for 1 h at room temperature with the following secondary antibodies: Alexa Fluor 488 donkey anti-mouse (Thermo Fisher Scientific), Alexa Fluor 488 donkey anti-rabbit (Thermo Fisher Scientific), Cy3 donkey anti-mouse (Thermo Fisher Scientific), Cy3 donkey anti-rabbit (Thermo Fisher Scientific). The slides were mounted with Vectashield mounting medium containing DAPI (H1200, Vector Labs). All antibodies are listed in Supplementary Table 2.

### Statistical Analysis

Statistical Analysis was carried out with the GraphPad Prism 6.0, Software Inc., USA and statistically significant differences were considered when the *p* < 0.05. ANOVA test (Tukey's *post-hoc* test) was applied for quantification of OU size and survival rate. Statistical significance was calculated with the student *t*-test for the OU proliferation assay, qRT-PCR, Western blots and TESI mass determination, and non-parametric Mann-Whitney test was applied for quantitative analysis of TESI complexity scores. In all analyses, the outliers were removed through the ROUT method.

## Results

### A. Organoid Unit *in vitro*

Throughout the culture period, OU growth, and survival rates were evaluated and cells were harvested on day 3 for analysis of target genes expression at the mRNA (qRT-PCR) and protein (Immunofluorescence and Western blot) levels. The results obtained are presented below and the detailed methodology may be found in the Materials and Methods section.

#### RSPO1 Treatment Increases the Size and Survival Rate of OU *in vitro*

**Figure 1:**
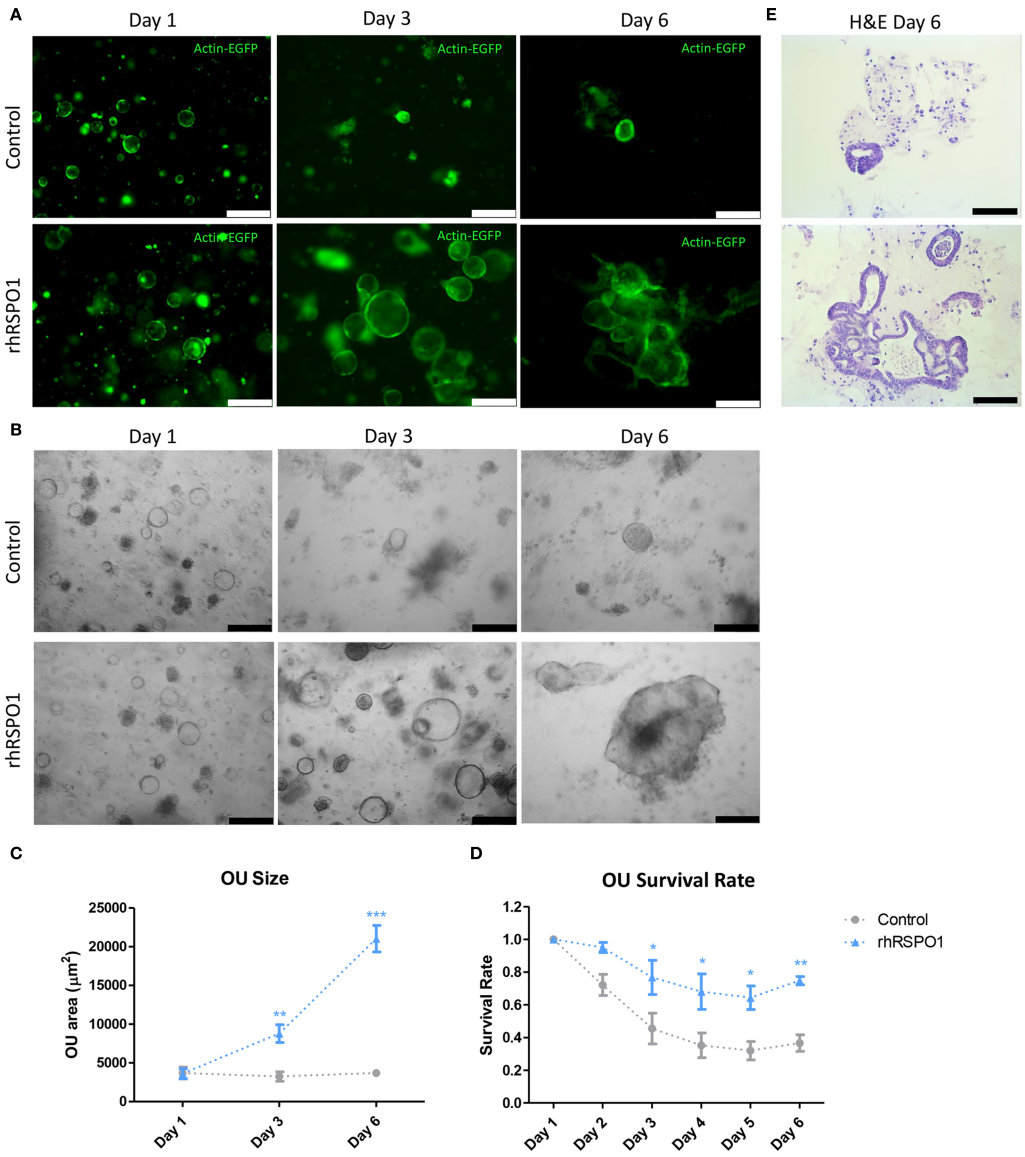
OU growth and survival *in vitro*. Fluorescence **(A)** or bright field **(B)** images of actin^GFP^ intestinal OU after 1, 3, and 6 days of treatment with rhRSPO1 (500 ng/mL) or PBS (control) in culture. **(C)** rhRSPO1-treated OU presented a larger area compared to the control group after 3 days, becoming 5.7 times larger on day 6. **(D)** Increased survival was observed from the third day of culture on, representing a 2-fold increase in OU survival between days 3 and 6 days in culture. **(E)** Day 6 OU H&E staining. ANOVA test (Tukey's *post-hoc* test) was carried out for quantification of OU size and survival rate. *p < 0.05; **p < 0.005; and ***p < 0.0005. Scale bars of **(A,B)** correspond to 250 μm, while scale bars of **(E)** is equivalent to 100 μm.

Treatment of intestinal organoid units with rhRSPO1 *in vitro* resulted in an increase in their size and survival rate. After 3 days of culture, the size of the rhRSPO1-treated (500 ng/mL) OU was 2.7 times that of the control group (8784.9 μm^2^ ± 1148.35 SEM vs. 3239.13 μm^2^ ± 601.71 SEM, *p* < 0.01) and 5.7 times larger on day 6 (21033.03 μm^2^ ± 1714.65 SEM vs. 3681.85 μm^2^ ± 428.21 SEM, *p* < 0.001; Figures 1A–C). Additional images representing the OU growth under different cultivation conditions (rhRSPO1 or PBS) and at different time points (Days 1, 3, and 6), for each independent experiment, may be found in Supplementary Figure 3. It is worth noting that the rhRSPO1-treated OU lost their perfect spherical shape after 6 days of treatment, becoming more complex three-dimensional structures, with projections and indentations, as may be observed in Figures 1A,B,E and Supplementary Figure 3. Additionally, an increase in survival was observed from the third day of culture on, representing a 2-fold increase in OU survival between day 3 (0.767 ± 0.104 SEM vs. 0.454 ± 0.094 SEM, *p* < 0.05) and 6 (0.747 ± 0.025 SEM vs. 0.365 ± 0.050 SEM, *p* < 0.01) in culture (Figure 1D). Histological analysis by H&E staining indicated the formation of OU with a simple columnar epithelium, containing a luminal region in its interior (Figure 1E).

#### RSPO1 Treatment Contributes to Maintenance of the Stem Cell Niche

**Figure 2:**
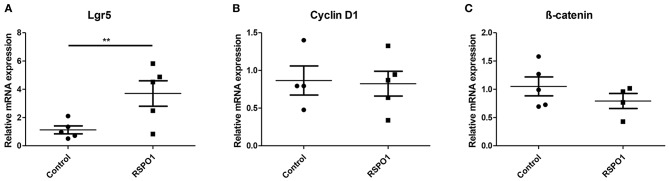
Relative mRNA expression of key Wnt signaling pathway genes in OU at Day 3 of rhRSPO1 (500 ng/mL) or PBS treatment, as assessed by qRT-PCR. **(A)** *Lgr5*; **(B)** *Cyclin D1*; **(C)** β*-catenin*. Student *t*-test was carried out for statistical analysis. **p < 0.005. Data were normalized relative to *Gapdh* as the reference housekeeping gene.

Lgr5 is known to be a stem cell marker and also the main receptor for the RSPO1 protein, acting through induction of the Wnt pathway. A 3.3 higher relative expression of *Lgr5* relative to the control group was found in the day 3 rhRSPO1-treated OU (3.70 ± 0.90 SEM vs. 1.128 ± 0.28 SEM, *p* = 0.009), indicating a higher relative prevalence of stem cells or a cellular adaptation to generate more Lgr5 receptor molecules (Figure 2A). No significant differences were observed in the relative expression levels of the cell cycle-related Wnt target gene, C*yclin D1* and of β*-catenin* (Figures 2B,C).

#### RSPO1 Treatment Activates the Beta-Catenin-Dependent Wnt Signaling Pathway

**Figure 3:**
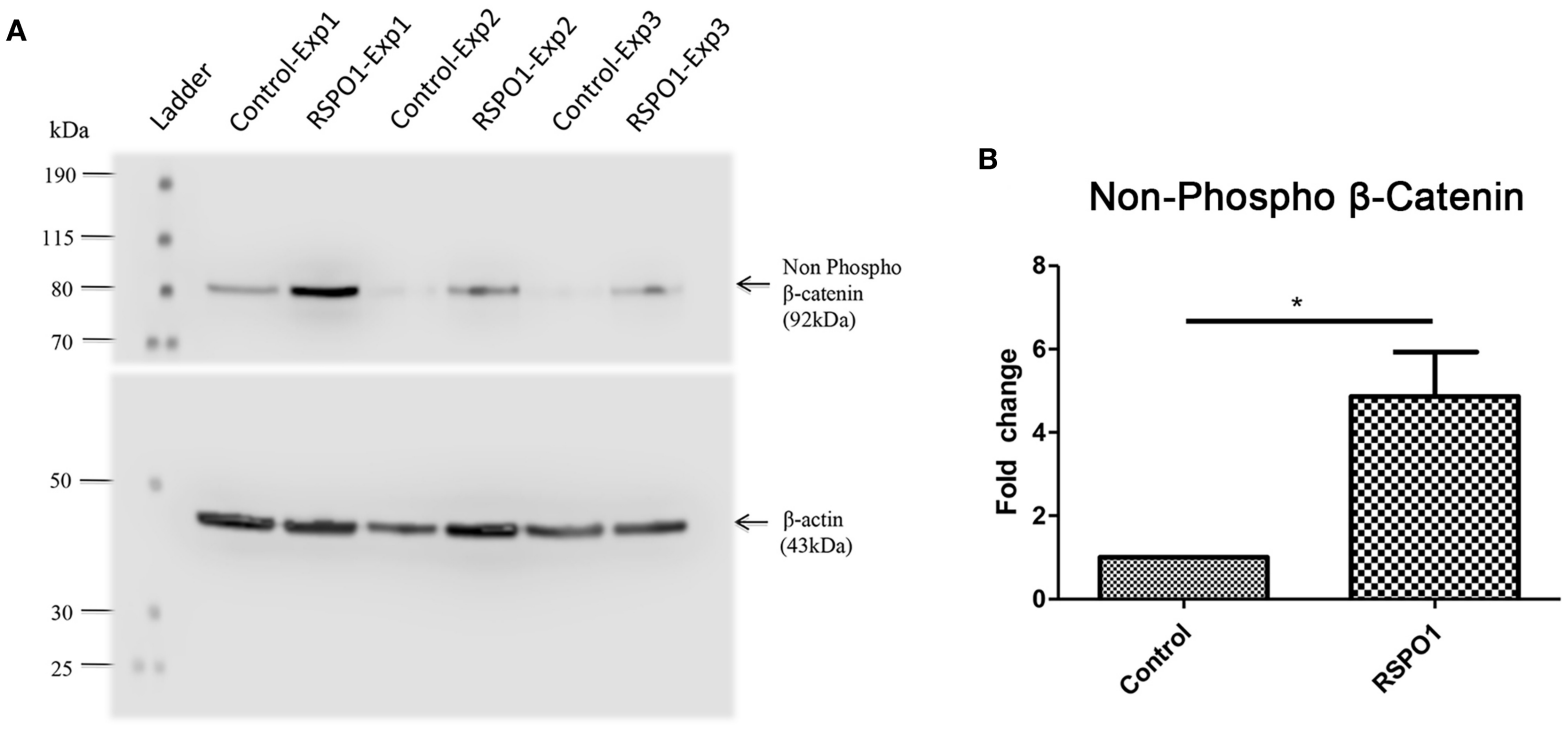
Relative protein expression of non-phosphorylated (active) β-catenin in OU at Day 3 of rhRSPO1 (500 ng/mL) or PBS treatment, as assessed by Western blot in three independent experiments. **(A)** Western blot analysis of non-phosphorylated (active) β-catenin: Ladder; Exp1 Control; Exp1 rhRSPO1; Exp2 Control; Exp2 rhRSPO1; Exp3 Control; Exp3 rhRSPO1. **(B)** WB densitometric analysis (quantification). Student *t*-test was carried out for statistical analysis. *p < 0.05.

The increase of non-phosphorylated (active) β-catenin in the day 3 rhRSPO1-treated OU group, observed by Western blot assay (Figure 3A), confirms the activation of the Wnt canonical pathway, also called the beta-catenin dependent pathway. Densitometric analysis of the Western blot membrane was carried out to quantify the amount active β-catenin, indicating a 4.8-fold increase compared to the control (4.865 ± 1.066 SEM vs. 1.000 ± 0.0 SEM, *p* = 0.034), after normalization relative to the β-actin reference protein (Figure 3B).

### B. Tissue Engineered Small Intestine Formation *in vivo*

**Figure 4:**
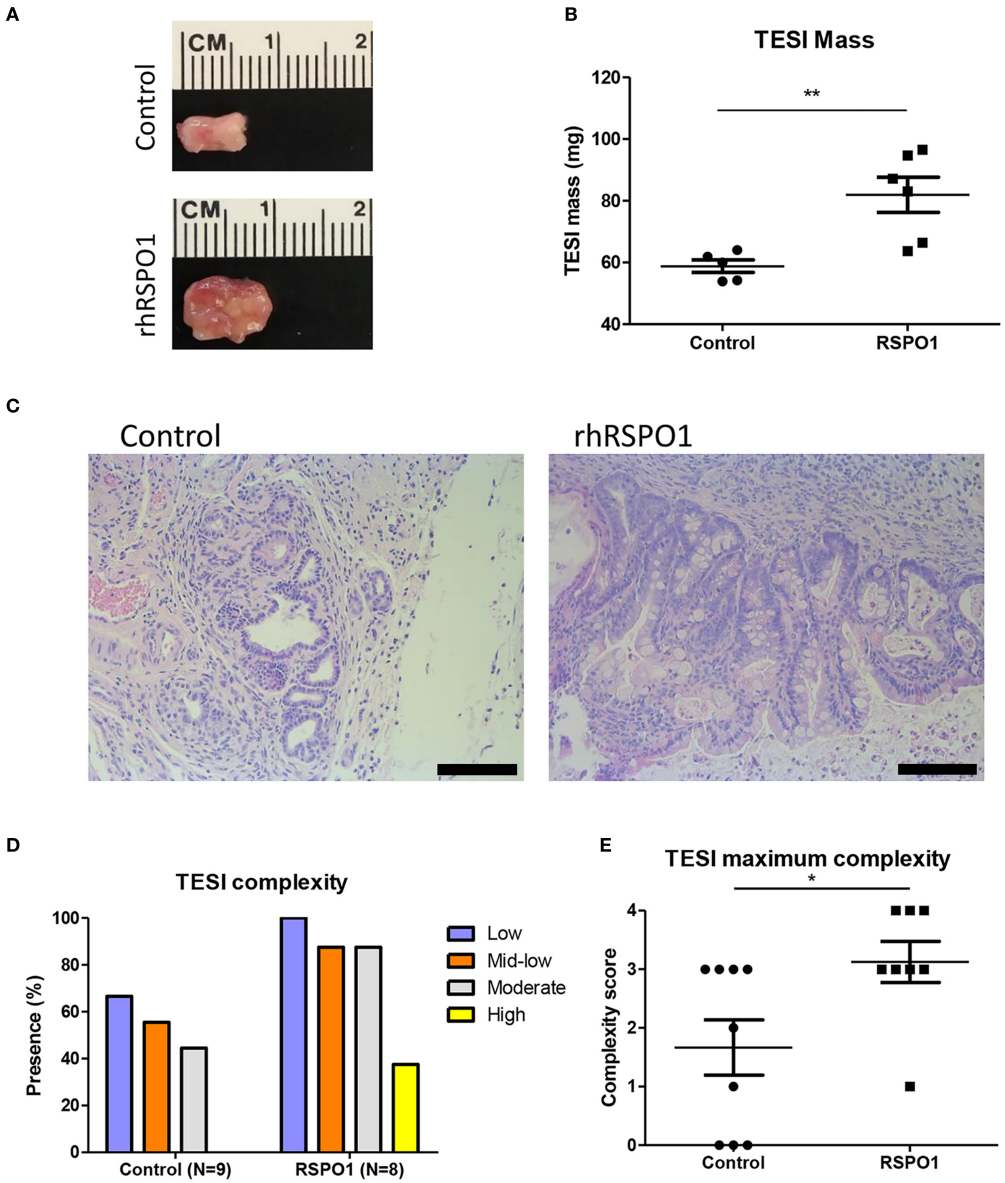
*In vivo* TESI development in biodegradable scaffolds in the presence and absence of rhRSPO1 upon explantation. **(A)** TESI explants after 1 month of *in vivo* maturation; **(B)** TESI mass after explantation; **(C)** TESI H&E staining; **(D)** TESI complexity; **(E)** TESI maximum complexity. TESI complexity scores 1–4 correspond to low, medium-low, moderate, or high complexity, respectively. *p < 0.05 and **p < 0.005. Scale bars correspond to 100 μm.

To evaluate the activity of rhRSPO1 in tissue engineered small intestine (TESI), mouse OU were seeded onto biodegradable scaffolds, in the presence or absence of rhRSPO1, and implanted into the omentum of NOD/SCID mice to generate TESI (Figure 4). The explants were harvested after 30 days, weighed, fixed in formalin and embedded in paraffin for histological and immunofluorescence analysis for different cell markers.

#### RSPO1 Treatment Increases TESI Size and Development *in vivo*

After 30 days of *in vivo* maturation, the explants which had been previously treated with rhRSPO1 displayed a larger size and mass than those treated with saline solution alone (81.93 mg ± 5.718 SEM vs. 58.80 mg ± 2.040 SEM, *p* = 0.0033; Figures 4A,B). Histological analysis of the engineered tissues and their respective degrees of intestinal epithelium complexity indicated that the scaffolds which were previously treated with rhRSPO1 before OU seeding developed a more mature intestinal epithelium, presenting well-formed villi and crypts (Figure 4C). The engineered tissues were blindly evaluated by three independent researchers, who consistently found a higher tissue organization complexity in the rhRSPO1-treated group, especially regarding the presence of mature crypts and villi (Figures 4D,E). In addition, the rhRSPO1-treated scaffolds generated TESI with some degree of organization in all eight implanted OU-loaded scaffolds (100%), whereas the control group (PBS) was able to develop TESI in only 6 out of 9 OU-loaded scaffolds (66.6%). No tumors or abnormal structures were found during animal necropsy and processing of TESI samples.

#### RSPO1-Treated TESI Forms a Completely Differentiated Mucosa at 1 Month

**Figure 5:**
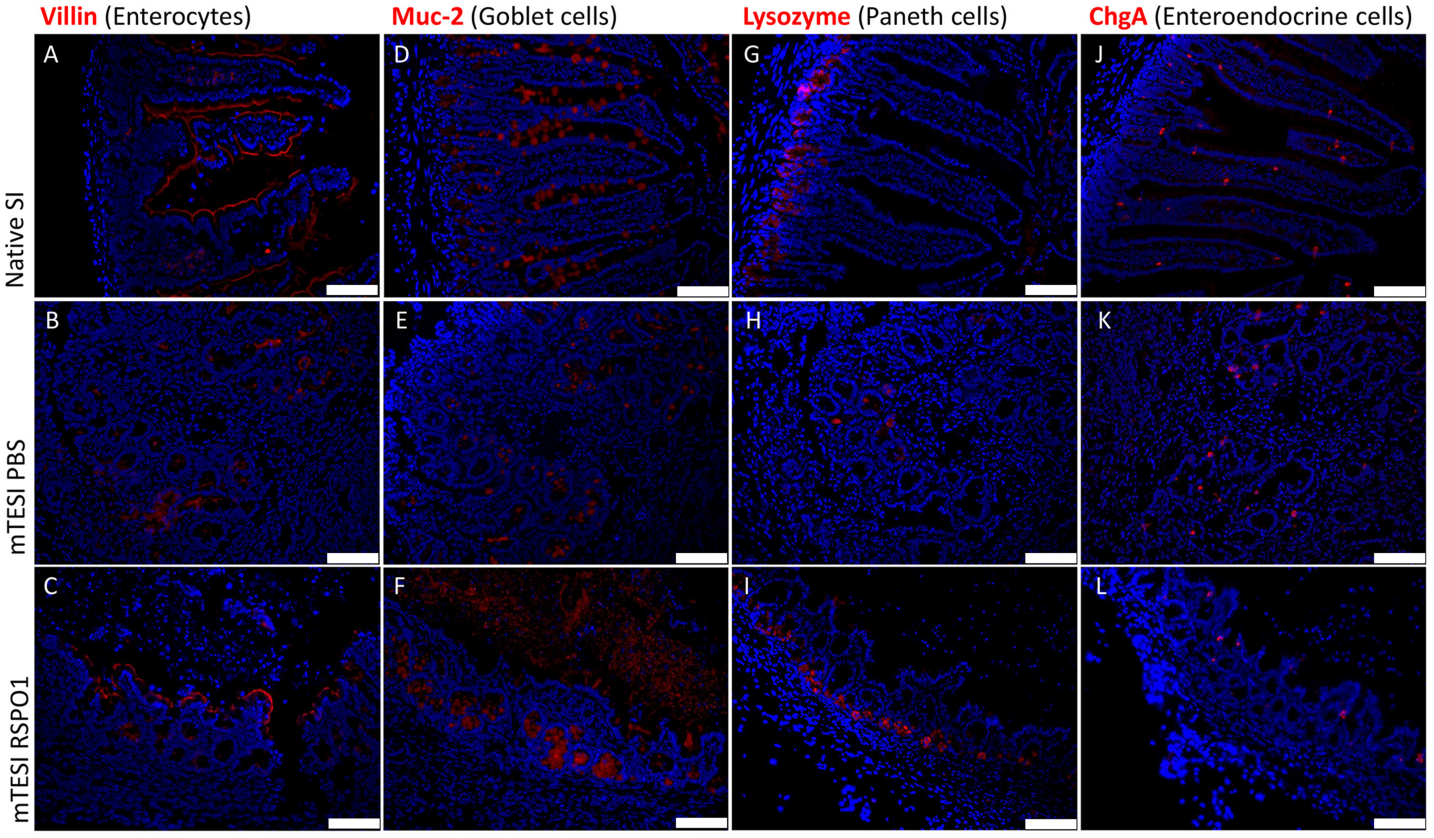
Immunofluorescent staining for differentiated intestinal epithelial cells found in rhRSPO1-treated TESI, as compared with native small intestine (SI) and TESI treated with PBS alone (control). Groups (lines): mouse native small intestine, mTESI PBS, mTESI rhRSPO1. Staining (red): Villin **(A–C)**, Muc-2 **(D–F)**, Lysozyme **(G–I)**, and ChgA **(J–L)** proteins. Scale bars correspond to 100 μm.

The TESI epithelium was further qualitatively characterized in terms of its structure and composition through specific markers for each of the differentiated cell types of the intestinal epithelium by immunofluorescence. As may be seen in Figure 5 (staining in red), the generated TESI showed all major cell types of the intestinal epithelium, namely: enterocytes (Villin^+^ absorptive cells), goblet cells (Muc-2^+^ mucus secreting cells), Paneth cells (Lysozyme^+^ stem cells niche maintainer cells), and enteroendocrine cells (ChgA^+^ hormone-secreting cells). The epithelial lining of TESI exposed to rhRSPO1 is completely covered with enterocytes, as may be observed by staining for Villin (Figures 5A–C), a protein associated with the brush border structure present in the intestine. In addition, it was possible to identify the presence of Goblet cells along the crypt-villus axis, as well as the presence of mucin in the luminal region stained with a Muc-2 antibody (Figures 5D–F). Paneth cells, stained with a lysozyme antibody, are responsible for the secretion of important factors for maintenance of the stem cell niche and are located at the bottom of the crypt where the intestinal stem cells reside (Figures 5G–I). The enteroendocrine hormone-secreting cells are located along the villi, identified by chromogranin A^+^ (ChgA) staining (Figures 5J–L).

#### RSPO1-Treated TESI Displays an Intact Stem Cell Niche and an Innervated Muscular Layer

**Figure 6:**
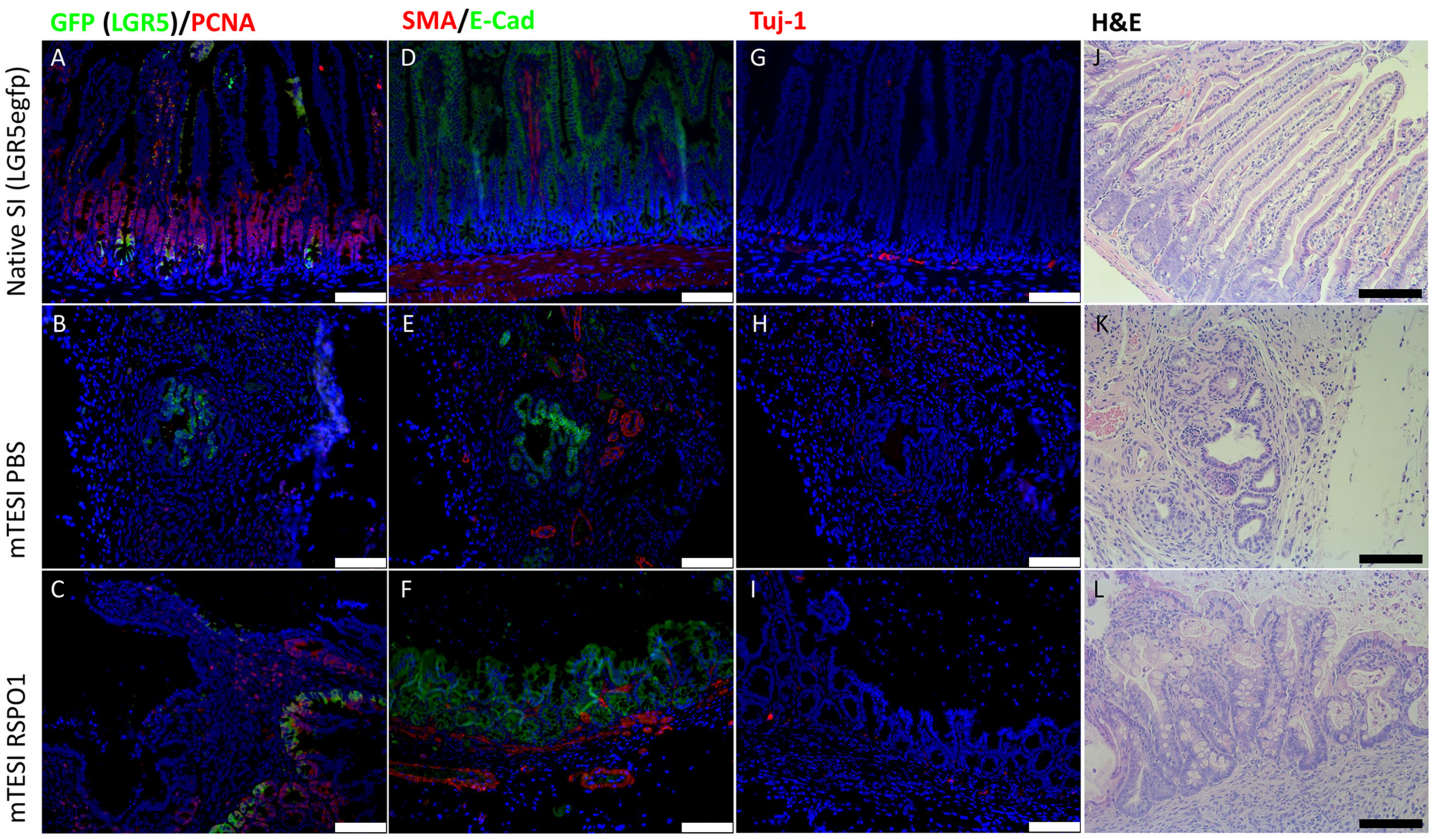
rhRSPO1-generated TESI have intestinal epithelial stem cells, a muscular layer and neuronal ganglia (Immunofluorescence). Groups (lines): mouse native small intestine, mTESI PBS, mTESI rhRSPO1. Staining: GFP (green)/PCNA (red) **(A–C)**, SMA (red)/E-cadherin (green) **(D–F)**, Tuj-1 (red) **(G–I)**, H&E **(J–L)**. Scale bars correspond to 100 μm.

rhRSPO1-treated TESI displays both intestinal stem cells and proliferating cells, as well as a muscular layer and a neural network associated with the TESI epithelial layer (Figure 6). GFP^+^ cells (green) indicate the presence of Lgr5^+^ stem cells in the crypt base (Figures 6A–C), the same region populated by Paneth cells. These cells are succeeded by transit amplifying cells (TA), cells in the proliferative state detected by PCNA^+^ staining (red) (Figures 6A–C). The smooth muscle layer (red) is labeled by the expression of smooth muscle α-actin (SMA^+^) below the intestinal epithelium in green (E-cadherin) (Figures 6D–F). The presence of neuronal ganglia is identified by Tuj-1^+^ staining (red) (Figures 6G–I). Tissues were stained with H&E for comparative histological analysis (Figures 6J–L).

## Discussion

To create a functional and viable tissue-engineered small intestine, new approaches to improve TESI formation are necessary. Among the several challenges and difficulties to render this form of treatment feasible, the *in vitro* maintenance and expansion of intestinal OU for TESI generation with sufficient mass for transplantation is crucial (Belchior et al., 2014; Grant et al., 2015; Hou et al., 2018). Since rhRSPO1 displays mitogenic activity in intestinal stem cells and promotes enteroids culture *in vitro*, acting through activation of the Wnt pathway, we sought to overcome these bottlenecks by applying this protein toward intestinal OU culture and TESI formation.

Here, we show that mouse intestinal organoid units treated with recombinant human RSPO1 display increased size and survival *in vitro* (Figure 1), an improvement which may be useful for subsequent TESI generation, particularly in cases where the donor source material is limited. This effect of rhRSPO1 is due to its activation of the canonical Wnt/β-catenin pathway, evidenced by the increased non-phosphorylated (active) beta-catenin protein expression in rhRSPO1-treated OU (Figure 3). As expected, no differences in total β*-catenin* gene expression were observed, since activation of the Wnt pathway acts stabilizing β-catenin at the protein level. It is widely known that the active non-phosphorylated β-catenin protein is stabilized by activation of the Wnt pathway, accumulates in the cytoplasm of the cells and that its translocation into the nucleus is an essential step for activation of transcriptional factors and Wnt target genes which are responsible for triggering the proliferative response of the cells (Cadigan and Peifer, 2009; MacDonald et al., 2009; de Lau et al., 2012).

Although RSPO1 and Wnt ligands together induce stem cells proliferation, these proteins have different actions in the intestinal epithelium self-renewal process (Yan et al., 2017). It is known that RSPO1 acts as a mitogen in Lgr5+ intestinal stem cells (Pinto and Clevers, 2005), presenting a synergistic effect with Wnt proteins to potentiate the canonical Wnt/beta-catenin signaling pathway (Kazanskaya et al., 2004; Kim et al., 2005). Wnt binds to Fzd receptors to activate this pathway, whereas RSPO1 binds to leucine-rich repeat containing G protein-coupled receptors 4-6 (Lgr4-6) potentiating their effect. RSPO1-Lgr4-6 binding increases the turnover of Wnt receptors through ZNRF3 protein inhibition, consequently increasing the availability of these receptors in the membrane (Carmon et al., 2011; de Lau et al., 2011; Glinka et al., 2011; Hao et al., 2012; Schuijers and Clevers, 2012). Therefore, since Lgr5 is a stem cell marker, as well as the major RSPO1 receptor, the increased *Lgr5* relative expression (Figure 2) in the rhRSPO1-treated OU group, compared with the control group, suggests either an increase in the relative concentration of stem cells or in the production of these receptors. In both cases, the results suggest that treatment with rhRSPO1 promotes both maintenance of the OU stem cell niche and their stemness.

Several reports in the literature have shown that it is possible to form TESI from organoid units of different organisms, including murine (Grikscheit et al., 2004; Sala et al., 2011; Grant et al., 2015; Hou et al., 2018), porcine (Sala et al., 2009), and human (Levin et al., 2013; Finkbeiner et al., 2015; Grant et al., 2015; Hou et al., 2018), seeded onto polymer scaffolds. Although it has been possible to improve SBS symptoms in Lewis rats by implanting TESI *in vivo* (Grikscheit et al., 2004), so far, this technology has not been tested to rescue the SBS symptoms in other species, such as mice and humans, despite the fact that digestive, and absorptive functions have been demonstrated in these models (Grant et al., 2015). Therefore, we generated mouse TESI (mTESI) from rhRSPO1-treated OU, in order to optimize TESI formation and to improve the amounts of starting material and resulting tissue-engineered intestine. We observed the formation of TESI in both the control (PBS) and rhRSPO1-treated groups, but with different degrees of efficiency and maturity. A clear improvement was observed in TESI formation in the presence of rhRSPO1, evidenced by an increased engraftment success rate, larger TESI mass and greater level of development, relative to the control group (Figure 4).

Addition of rhRSPO1 to TESI significantly increased its efficiency, leading to formation of a tissue-engineered intestinal epithelium in 100% of the samples (*N* = 8), compared to 66.6% in the control group (*N* = 9). In addition, ~40% (3/8) of these TESI reached the highest degree of development (high complexity), presenting an intestinal epithelium containing mature villi, crypts and well-developed secretory cells. Although we did not observe a mature TESI (high complexity) in the control group, maintained in the absence of rhRSPO1, previous results from our group demonstrated that it is possible to achieve a fully mature mTESI in the absence of exogenous peptide growth factors (Sala et al., 2011), although at lower efficiency, underscoring the need for optimization of this process.

In order to ensure TESI function, it is important to maintain the correct structure present in the native intestine. Therefore, the engineered tissues were qualitatively characterized relative to the cellular components present in the intestinal epithelium, as well as components of the enteric nervous system and associated mesenchymal structures, including the muscle layer. Specific cell markers indicated the formation of a mature tissue-engineered intestinal epithelium containing all of the differentiated cell types at their respective sites, with the presence of fully polarized enterocytes lining epithelium, goblet cells along the entire crypt-villus axis, Paneth cells at the base of the crypt, and enteroendocrine cells along the villi (Figure 5). The enterocytes, which cover the epithelium with their brush-border, ensure a good absorptive surface, while goblet cells secrete mucus to the lumen. The enteroendocrine cells secrete hormones, which regulate intestinal functioning, controlling peristalsis, appetite, secretory function of other glands, and other physiological processes. Paneth cells are responsible for the secretion of various peptide factors at the base of the crypt, conditioning their environment to maintain the stem cell niche (Day, 2006; Potten et al., 2009; van der Flier and Clevers, 2009; Sato et al., 2011).

In addition to fully differentiated cells, the rhRSPO1-induced TESI epithelium also displayed Lgr5^+^ stem cells at the base of the crypt and a transit-amplifying cells (TA) region, with a high proliferative rate in the crypt-villus transition zone (Figure 6), which is known to be important for promoting the intestinal self-renewal process (Potten et al., 2009; Schuijers and Clevers, 2012). As in the native intestine, the presence of a muscular layer and neuronal ganglia, located below the intestinal epithelium, was also observed in the rhRSPO1-induced TESI (Figure 6).

Although Zhao et al. argue that RSPO1 treatment can reduce the tumorigenic risk by decreasing inflammation in inflammatory bowel disease (Zhao et al., 2007), several authors suggest that aberrant Wnt pathway signaling, especially hyperactivation, is strongly associated with the presence of various types of cancer in humans and mice, including colon cancer (Yoon and Lee, 2012). Thus, although Abo and Clevers argue that the therapeutic use of RSPO1 is safer than WNT pathway ligands (Abo and Clevers, 2012), attention should be paid to the possibility of developing tumors resulting from the therapeutic use of rhRSPO1. Therefore, it is important to note that, in the present study, no tumors were found during animal necropsy or processing of TESI samples. However, further and longer studies are required to ensure the safety of this rhRSPO1 therapy.

## Conclusion

In conclusion, treatment with recombinant human RSPO1 and consequent activation of the Wnt canonical pathway improves the culture of mouse intestinal organoid units (OU) *in vitro*, increasing their size and survival rate. In addition, treatment of scaffolds and OU with rhRSPO1 improved mTESI formation *in vivo*, increasing its mass, development, and engraftment success rate. This evolution of the TESI protocol allowed efficient generation of well-developed TESI, containing a mature tissue-engineered intestinal epithelium with crypt and villus structures, composed of absorptive cells, secretory cells, enteroendocrine cells, Paneth cells, TA cells, and stem-cells, properly surrounded by an innervated muscularis. Therefore, the treatment with rhRSPO1 represents an important advance in optimization of the murine TESI formation process, indicating that, in the future, this protein may also improve the clinical viability of this therapy for treatment of SBS patients.

## Data Availability Statement

All datasets generated for this study are included in the article/Supplementary Material.

## Ethics Statement

This animal study was reviewed and approved by IACUC of Children's Hospital Los Angeles.

## Author Contributions

GL contributed to the design of the study, executed the experiments, analyzed the data, and wrote the manuscript. SZ contributed to the design of the study, carried out some *in vivo* experiments, and analyzed the data. AS performed some of the *in vivo* experiments and contributed to data analysis. TG, MS, and AC contributed to the study design, data analysis, manuscript writing, and editorial revisions. All authors approved the final submitted version of the article.

## Conflict of Interest

The authors declare that the research was conducted in the absence of any commercial or financial relationships that could be construed as a potential conflict of interest.
