# Supplementary material for: R-Spondin 1 (RSPO1) Increases Mouse Intestinal Organoid Unit Size and Survival in vitro and Improves Tissue-Engineered Small Intestine Formation in vivo
Source: Front Bioeng Biotechnol. 2020 Jun 5;8:476. doi: 10.3389/fbioe.2020.00476 (PMC7295003; doi:10.3389/fbioe.2020.00476)
Supplement: Supplementary file 1 [file Table_1.doc]

Supplementary Material

**Supplementary Table 1.** Primers list.

| *Primer* | Concentration (nM) | Forward Sequence | Reverse Sequence |
| --- | --- | --- | --- |
| *Cyclin D1* | 600 | CAGAAGTGCGAAGAGGAGGTC | TCATCTTAGAGGCCACGAACAT |
| *β-Catenin* | 600 | CCCAGTCCTTCACGCAAGAG | CATCTAGCGTCTCAGGGAACA |
| *Lgr5* | 600 | CTTCACTCGGTGCAGTGCT | CAGCCAGCTACCAAATAGGTG |
| *Gapdh* | 200 | CTCCCACTCTTCCACCTTCG | GCCTCTCTTGCTCAGTGTCC |
