# Supplementary material for: R-Spondin 1 (RSPO1) Increases Mouse Intestinal Organoid Unit Size and Survival in vitro and Improves Tissue-Engineered Small Intestine Formation in vivo
Source: Front Bioeng Biotechnol. 2020 Jun 5;8:476. doi: 10.3389/fbioe.2020.00476 (PMC7295003; doi:10.3389/fbioe.2020.00476)
Supplement: Supplementary file 2 [file Table_2.doc]

Supplementary Material

**Supplementary Table 2.** Antibodies list.

| **Primary Antibodies Target** | **Ab** | **Species** | **Dilution** | **Assay** | **Ref. N°.** | **Company** |
| --- | --- | --- | --- | --- | --- | --- |
| Non-phospho β-catenin | mAb | rabbit | 1:1,000 | WB | #8814 | Cell Signaling |
| β-actin | mAb | mouse | 1:1,000 | WB | sc-69879 | Santa Cruz |
| Chromogranin A | pAb | rabbit | 1:400 | IF | ab15160 | Abcam |
| E-Cadherin | mAb | mouse | 1:100 | IF | 610181 | BD |
| GFP | pAb | rabbit | 1:200 | IF | ab290 | Abcam |
| Lysozyme | pAb | rabbit | 1:100 | IF | A0099 | DAKO |
| Mucin2 | pAb | rabbit | 1:100 | IF | sc-15334 | Santa Cruz |
| PCNA | mAb | mouse | 1:400 | IF | ab29 | Abcam |
| Smooth Muscle D-Actin | pAb | rabbit | 1:100 | IF | ab5694 | Abcam |
| Tuj1 | mAb | mouse | 1:1,000 | IF | 801202 | BioLegend |
| Villin | mAb | mouse | 1:100 | IF | sc-66022 | Santa Cruz |
| **Secondary Antibodies** | **Ab** | **Species** | **Dilution** | **Assay** | **Ref N°** | **Company** |
| anti-mouse IgG HRP linked | pAb | goat | 1:1,000 | WB | #70746 | Cell Signaling |
| anti-rabbit IgG HRP linked | pAb | goat | 1:1,000 | WB | #70745 | Cell Signaling |
| anti-mouse Alexa Fluor 488 | pAb | donkey | 1:200 | IF | A-21202 | Thermo Fisher |
| anti-rabbit Alexa Fluor 488 | pAb | donkey | 1:200 | IF | A-21206 | Thermo Fisher |
| anti-mouse Alexa Fluor 555 | pAb | donkey | 1:200 | IF | A-31570 | Thermo Fisher |
| anti-rabbit Alexa Fluor 555 | pAb | donkey | 1:200 | IF | A-31572 | Thermo Fisher |
