# Supplementary material for: R-Spondin 1 (RSPO1) Increases Mouse Intestinal Organoid Unit Size and Survival in vitro and Improves Tissue-Engineered Small Intestine Formation in vivo
Source: Front Bioeng Biotechnol. 2020 Jun 5;8:476. doi: 10.3389/fbioe.2020.00476 (PMC7295003; doi:10.3389/fbioe.2020.00476)
Supplement: Supplementary file 3 [file Data_Sheet_1.doc]

Supplementary Material

## Supplementary Figures

**Supplementary Figure 1.** Preliminary rhRSPO1 dose standardization comparing two different concentrations (500ng/mL and 200ng/mL) in intestinal OU (A) growth and (B) survival response *in vitro.* PBS was used as the control. ANOVA test (Tukey’s post hoc test) was carried out for quantification of OU size and survival rate. * *p*<0.05; ** *p*<0.005; *** *p*<0.0005.

**Supplementary Figure 2.** TESI complexity based on H&E staining. (**A**) Low complexity – poorly developed epithelium in the form of rosettes; (**B**) Medium-low complexity - continuous linear epithelium; (**C**) Moderate complexity - continuous epithelium with villus-like and rudimentary crypts structures; (**D**) High complexity - continuous epithelium with well-defined crypts, villi and clear presence of secretory cells. Scale bars correspond to 100µm.

**Supplementary Figure 3.** Bright field images of intestinal OU growth *in vitro* after 1, 3 and 6 days of treatment with rhRSPO1 (500ng/mL) or PBS (control) in three independent experiments each of which with two technical replicates. Scale bar corresponds to 250µm and all images were acquired with the same magnification under optical microscope.
