# Supplementary figures and images for: R-Spondin 1 (RSPO1) Increases Mouse Intestinal Organoid Unit Size and Survival in vitro and Improves Tissue-Engineered Small Intestine Formation in vivo
Source: Front Bioeng Biotechnol. 2020 Jun 5;8:476. doi: 10.3389/fbioe.2020.00476 (PMC7295003; doi:10.3389/fbioe.2020.00476)

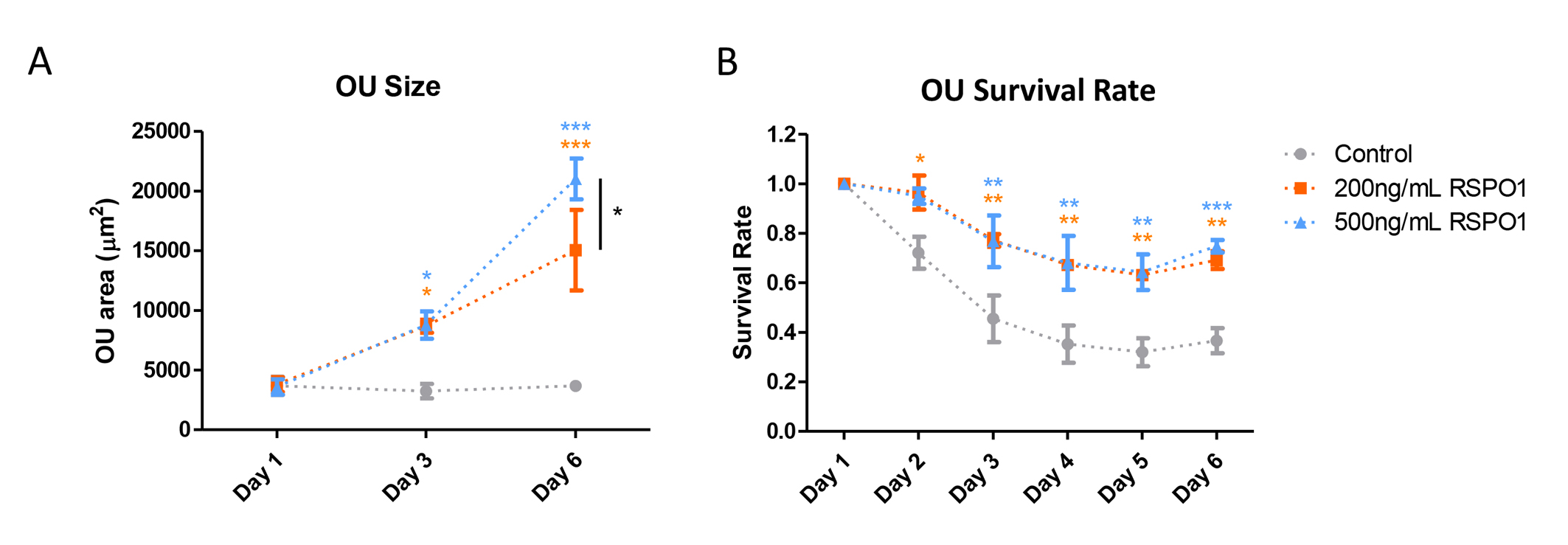

Supplement: Supplementary file 4 [file Image_1.JPEG]

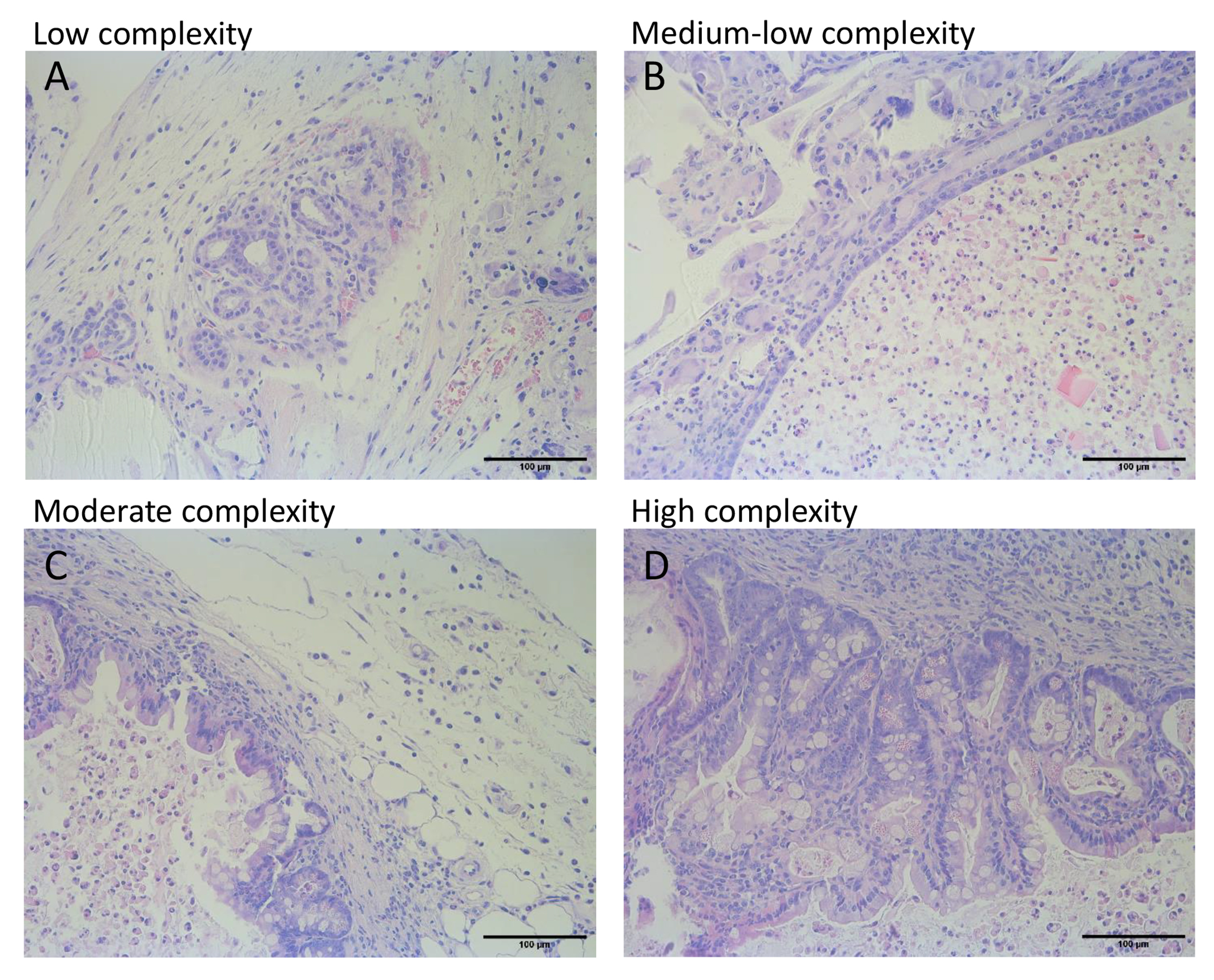

Supplement: Supplementary file 5 [file Image_2.JPEG]

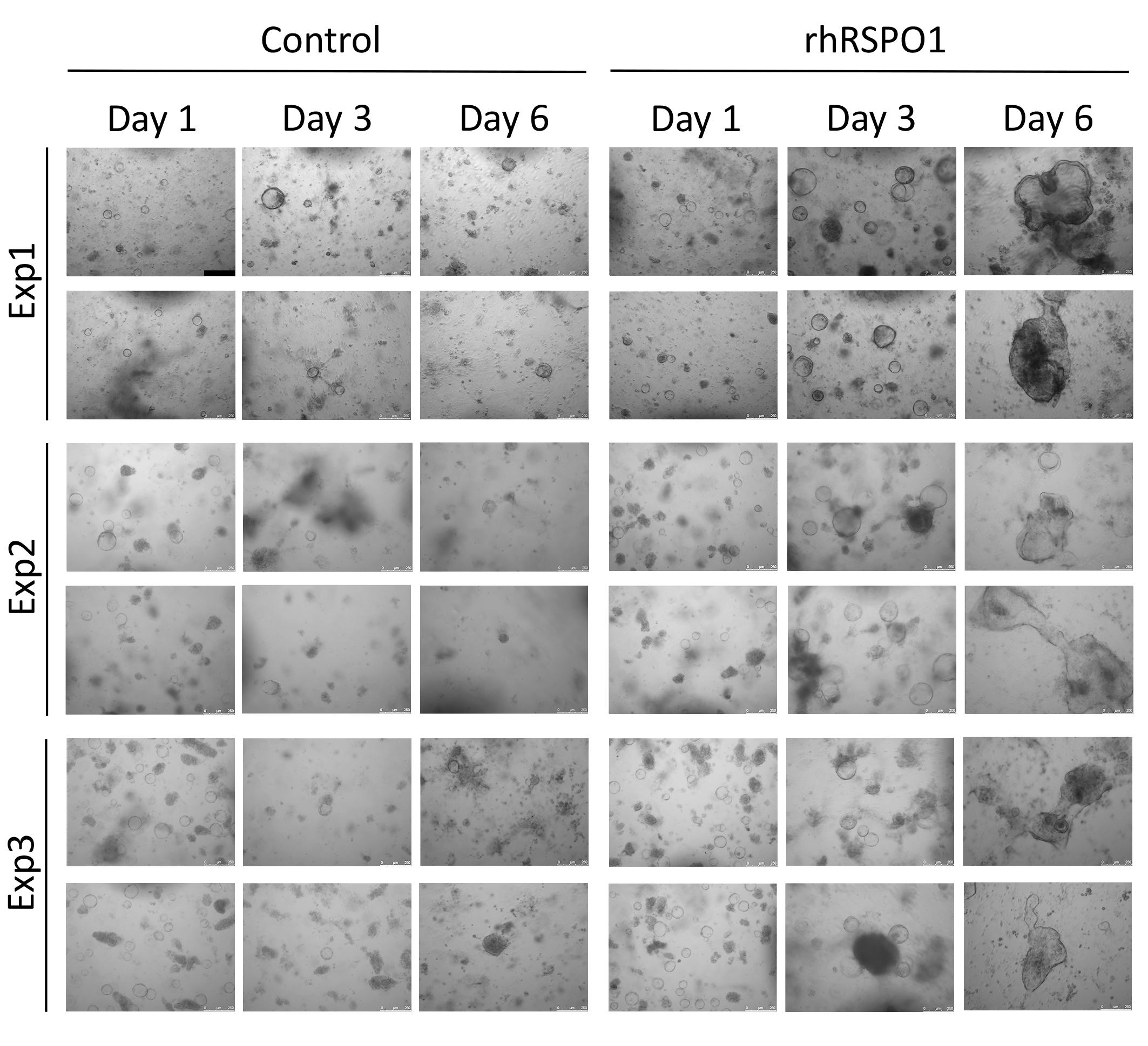

Supplement: Supplementary file 6 [file Image_3.JPEG]
